# Effect of dietary intervention treatment on children with iron deficiency anemia in China: a meta-analysis

**DOI:** 10.1186/s12944-018-0749-x

**Published:** 2018-05-10

**Authors:** Jian Sun, Lei Zhang, Jing Cui, Shanshan Li, Hongting Lu, Yong Zhang, Haiming Li, Jianping Sun, Zulqarnain Baloch

**Affiliations:** 1 Qingdao Women and Children’s Hospital, Qingdao, 266003 China; 2 The Affiliated Hospital of Qingdao University, Qingdao, China; 3 Qingdao Municipal Center for Disease Control and Prevention, Qingdao, China; 4 Qingdao Institute of Preventive medicine, Qingdao, China; 5 Sch Humanities and Law, South China Agricultural University, Guangzhou, 510642 China; 6 School of Public Health of Qingdao University, Qingdao, 266021 China; 7 College of Veterinary Medicine, South China Agricultural University, Guangzhou, 510642 China

**Keywords:** Meta-analysis, Iron deficiency anemia, Children, Dietary intervention, China

## Abstract

**Background:**

Previous studies have shown beneficial effects of dietary approaches for iron deficiency anemia (IDA) control. This study was design to investigate the effect of dietary intervention treatment on children with iron deficiency anemia.

**Methods:**

We performed a systematic review of published dietary interventions effect on IDA treatment through meta-analysis. CBM, CNKI, Wanfang database, EMBASE, VIP, PubMed and Web of science database were searched to identify studies published between January, 1980 and December, 2016. Statistical analysis was performed by Revmen5.2 software.

**Results:**

Initially we retrieved for 373 studies, and then 6 studies with a total of 676 individuals were included in the analysis according to the inclusion and exclusion criteria for meta-analysis. The overall pooled estimate of odds ratio [(OR), 95% confidence intervals (95% CI)] in the dietary intervention on children with iron deficiency anemia was 6.54 (95% CI: 3.48-12.31, Z = 5.82, *p*<0.001) and funnel plot is symmetric.

**Conclusions:**

Our meta-analysis suggested that dietary interventions are effective in improving the iron deficiency in children with iron deficiency anemia (IDA) and should be considered in the overall strategy of IDA management.

## Background

Iron deficiency anemia (IDA) is an anemia caused by a lack of iron which is a most common and widespread nutritional disorders among infants and children in the world particularly in under-developing countries [1]. It has been estimated that prevalence of IDA is very high in India (60% to 80%) than anywhere else in the world [2–6]. The prevalence of IDA is varying from 10 to 40% in P. R. China. Young children are at high risk for iron deficiency anemia due to their high dietary iron requirements. It has already been highlighted that IDA has a significant impact on motor, cognitive and socioemotional development, and is not reversible in children [7–9].

Iron is an important mineral that performs many biological functions in our body. Iron requirements is very high in younger age due to rapid growth with sharp increase in lean body mass, red cell mass and blood volume which raises iron requirements for hemoglobin in the blood and myoglobin in the muscle. However, failure to fulfil high iron demand leads to iron deficiency anemia. Additionally, high iron intake can also cause to health problem. In children with a genetic predisposition to absorb more iron than normal, therefore, iron may accumulate in body tissues over many decades, which may lead to tissue and organ damage [10, 11]. As IDA is a very common disease in the society and people normally prefer take oral iron supplementation therefore, side-effects of oral allopathic iron preparations are very frequently encountered. So, alternative and complementary treatment for IDA had been suggested, such as lifestyle modifications, especially dietary intervention. Diet is one of the main modifiable factors to recover normal iron level in IDA effected children, and additionally, dietary intervention as part of lifestyle intervention also improves iron deficiency status in children with IDA. Increasing evidence indicates that dietary intervention, assisting the treatment of IDA in children, can also improve the clinical effect.

Employing meta-analyses for the identification and analysis of findings from observational studies, one will synthesize research results that are needed by health care professionals and policy makers and provide them with important information on epidemiological indicator [12, 13]. Additionally, in meta-analysis sample size is increased as the studies are combined, resulting in a better statistical power. Meta-analysis can also explore the observed heterogeneity among the results of individual studies [14]. In our study, we take full advantage of the meta-analysis to establish the current evidence concerning the relationship between dietary intervention and IDA in children. It is necessary for a more comprehensive study on subject to sum up the available findings in the literature.

## Methods

### Strategy of literature search

The available articles publish in English or Chinese (up to December 2016) were identified by extended computer-based searches from the following databases: (1) Pubmed; (2) CNKI (National Knowledge infrastructure); (3) WanFang Med Online and (4) CBM (Chinese Biology Medical Literature Database). In order to maximize the sensitivity of the search, general keywords such as (iron deficiency anemia) and (dietary intervention) and (children or infant or adolescent) plus China were co-searched. We also reviewed the references cited in the studies and review articles to identify additional studies not captured by our database searches.

### Inclusion criteria and quality assessment

Two reviewers independently reviewed all the resultant titles and abstracts for IDA in children. The full-texts of all relevant articles were then assessed by either reviewer. Studies were independently selected for inclusion and were included in the study if they were diagnosed with iron deficiency anemia in children according to the standard.

The object of study: children with IDA using diagnostic criterion of China for IDA.

Study design: prospective randomized controlled study by published.

Intervention measures: children of IDA were randomly divided into treatment group and control group, children in control group treating with routine dose iron agent; and treatment group taken on the basins of drug therapy dietary intervention. Dietary intervention is mainly to eat about 30~ 40 g of pork liver, sheep liver, chicken liver, or 1~ 2 egg, or 30 red dates etc. before or after the meal once a day.

Overall assessment of result: clinical recovery—clinical symptoms disappeared completely, and hemoglobin returned to normal. Clinically effective—clinical symptoms relieved, and the rise of Hb>15 g/L^− 1^. Invalid—clinical symptoms didn’t improve or obviously improve, and the rise of Hb < 15 g/L^− 1^.

Studies were excluded if they were not primary studies, duplicated publication, and so on. Any disagreement between two reviewers was assessed by a third reviewer.

### Material selection and extraction

The selection of all studies and data extraction of eligible studies was both performed independently by 2 authors (ZB and JC). A consultation was performed with third party when a dispute occurred. All the three researchers are good at clinical epidemiological methodology and related domain knowledge. Extracting information Excel spreadsheet and fetching information: ① The general information: title, the first author, publish time and region. ② The characteristics of research: research type, the number of the case and the control group, the crowd source and distribution proportion of boy and girl. ③ Data characteristics: capacity for IDA, unit, relative risk (RR) and 95% confidence interval or odds ratio (OR) and 95% confidence interval. If data did not give within the literature, by statistical software got.

### Statistical analysis

The data were collected, analyzed and checked in accordance with the requirements of Meta-analysis. Statistical analysis was performed by Revmen5.2 software, which was provided by the Cochran Collaboration. If there was no statistically significant heterogeneity in this meta-analysis, the fixed effect model was employed. And the random effects model was used for meta-analysis when the results of trials had heterogeneity. The odds ratio (OR) was calculated for dichotomous data with 95% confidence intervals (CIs) for all analyses. *p* values that were *p<0.05* considered statistically significant. If necessary, we could use sensitivity analysis to test the stability of the results. Funnel-plot analysis was used to identify the publication bias.

## Results

### Characteristics of studies

**Fig. 1:**
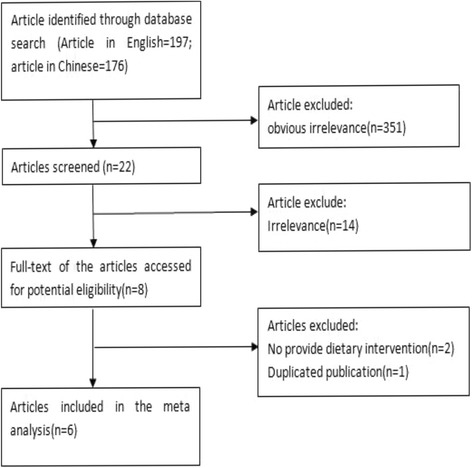
Flow char diagram of participants

We identified 6 articles with 8 eligible outcomes for this meta-analysis [15–20]. All articles were randomized control trials. Having assessed the quality of the full-texts of potentially relevant studies, 6 studies with a total of 676 individuals were included in the present systematic review. Figure 1 presented the flow chart for exclusion /inclusion process. The detailed information of the included studies from meta-analysis is listed in Table 1.

**Table 1:** The characteristics in studies included in this meta-analysis

Author	Year	Randomization	Blinding	Study field	Age	Case number		Intervention time	Intervention type and dosing		Reference
						Treatment	Control		Treatment	Control	
Wang SH	2008	Semi randomization	Unclear	Anyuan county Jiangsu province	1~ 13 years	50	49	4 weeks	Shengxuening tablet (0.5 g/tid po) + dietary intervetion (qd)	Shengxuening tablet (0.5 g/tid po)	[15]
Liu M	2010	Unclear	Unclear	Neijiang Sichuan province	2~ 6.5 years	75	75	28 days	Shengxuening tablet (0.5 g/tid po) + dietary intervetion (qd)	Shengxuening tablet (0.5 g/tid po)	[16]
Tian J	2010	Randomization	Unclear	Xuzhou Jiangsu province	9 months~ 11 years	60	60	2 months	Elemental iron (1 mg/kg bid po) + Vc(0.1 g/tid po) + dietary intervetion (qd)	Elemental iron (1 mg/kg bid po) + Vc(0.1 g/tid po)	[17]
Zhu M	2011	Randomization	Unclear	Xuzhou Jiangsu province	11 months~ 10 years	32	32	2 months	Elemental iron (1 mg/kg bid po) + Vc(0.1 g/tid po) + dietary intervetion (NA)	Elemental iron (2 mg/kg tid po) + Vc(0.1 g/tid po)	[18]
Qi GJ	2011	Randomization	Unclear	Xuzhou Jiangsu province	8 months~ 12 years	70	70	8 weeks	Elemental iron (1 mg/kg bid po) + Vc(0.1 g/tid po) + dietary intervetion (qd)	Elemental iron (2 mg/kg tid po) + Vc(0.1 g/tid po)	[19]
Lan H	2012	Randomization	Unclear	Pengzhou Jiangsu province	6 months~ 9 years	52	51	2 months	Elemental iron (1 mg/kg bid po) + Vc(0.1 g/tid po) + dietary intervetion(qd)	Elemental iron (2 mg/kg tid po) + Vc(0.1 g/tid po)	[20]

### Effect of dietary intervention on iron deficiency anemia

**Fig. 2:**
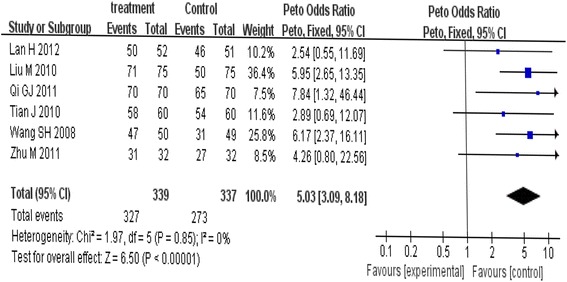
Meta-analysis of the effect of dietary intervention on children with IDA in fixed effect model

The change and the corresponding 95% CIs for iron deficiency anemia in children in each trial and overall are presented in Fig. 2. Compared with no intervention (control), dietary intervention was associated with an average change in clinical effective from 94.0% to 100.0%. The clinical effect was increased in dietary intervention in all the six trials, among which three trials had statistical increase of clinical effect. Tests for heterogeneity showed no significant differences across studies (χ2 = 1.97, *P* = 0.85), thus the fixed effect model was employ. The overall pooled estimate of OR in the dietary intervention on children with iron deficiency anemia was 5.03 (95% CI: 3.09-8.18, Z = 6.50, *P*<0.001).

### Results of subgroup analysis

#### Subgroup analysis for duration of dietary intervention

**Fig. 3:**
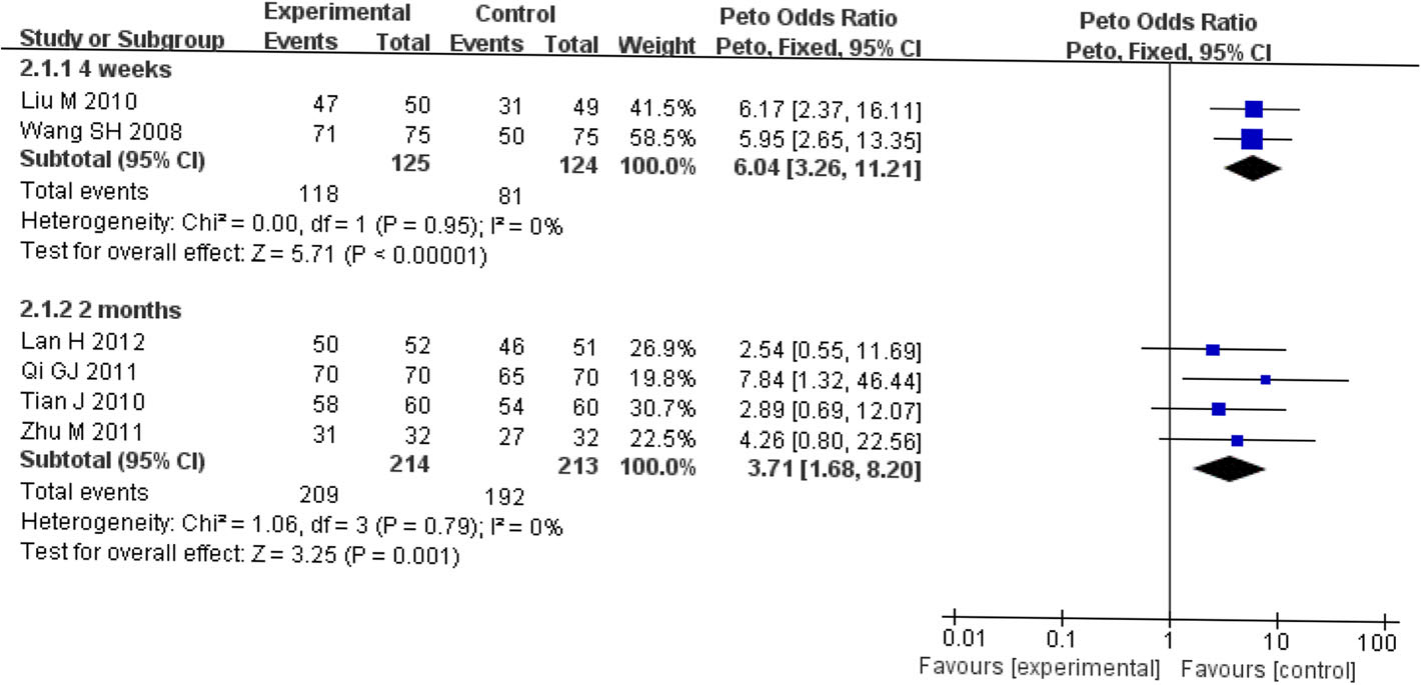
Stratified analysis of the effect of dietary intervention on children with IDA in random effects model

Subgroup analysis was conducted to investigate the effect of duration of intervention on the polled results. Tests for heterogeneity showed no significant differences across studies (4 weeks: χ2 = 0.00, *P* = 0.95; 2 months: χ2 = 1.06, *P* = 0.79), thus the fixed effect model was employ. OR of 4 weeks in the dietary intervention on children with iron deficiency anemia was 6.04 (95% CI: 3.26-11.21, Z = 5.71, *p*<0.0001*)*, and OR of 2 months was 3.71(95% CI: 1.68-8.20, Z = 3.25, *p* = 0.001). Duration of dietary intervention had no significant effect on OR. As shown in Table 2 and Fig. 3, subgroup analysis had not deduced any statistical effect on OR.

**Table 2:** Results of stratified analysis for the duration of dietary intervention

Group (duration of intervention)	Total data included	OR (95%CI)	*P*	P for heterogeneity	I^2^,%
4 weeks	2	6.04 (3.26, 11.21)	*<0.00001*	0.95	0
2 months	4	3.71 (1.68, 8.20)	*0.001*	0.79	0
All	6	5.03 (3.09,8.18)	*<0.00001*	0.85	0

#### Funnel-plot analysis

**Fig. 4:**
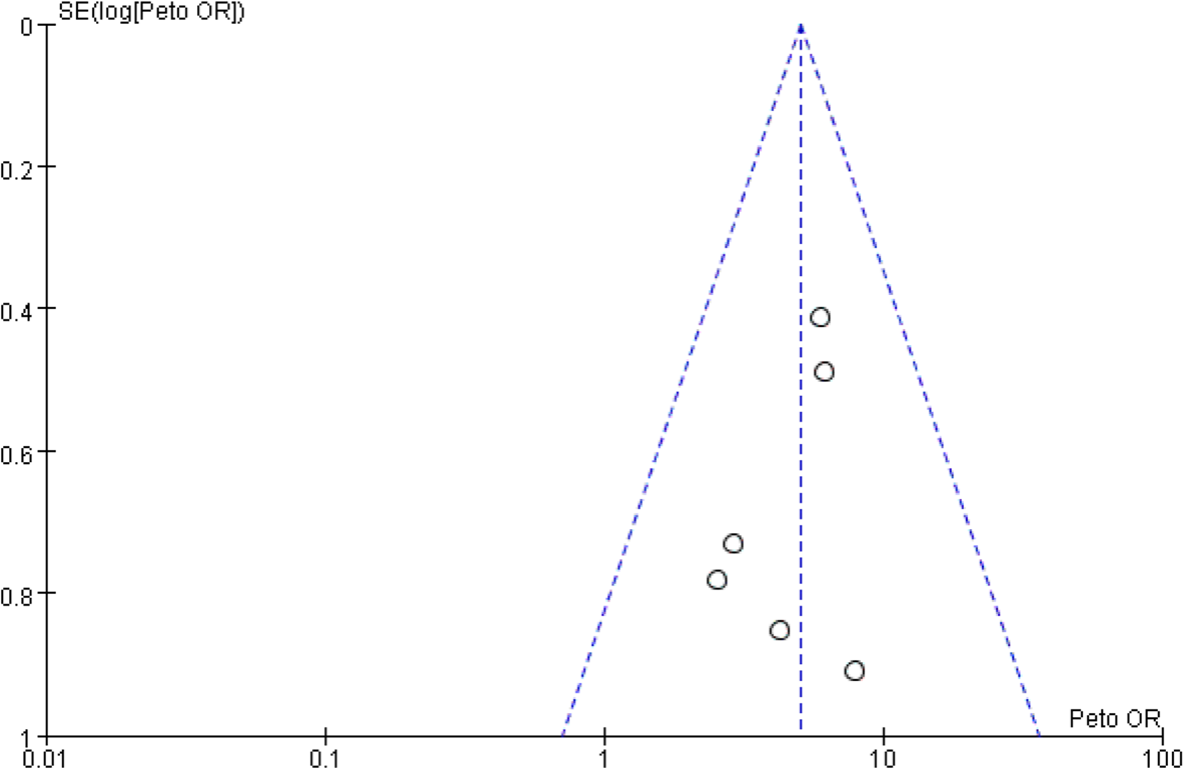
Funnel-plot of random control trials

Using STATA software to analyze the publication bias for the 10 articles, Begg’s test shows that *p* value is higher than 0.05, indicating that there are no significant publication bias was observed in the selected studies (Begg’s funnel plot was symmetric Fig. 4).

#### Sensitivity analysis

In the sensitivity analyses, while analysis using random effect model showed that no trials had substantial influence on the pooled analysis. The results are basically similar in the two models.

## Discussion

Overall Anemia is a global health problem of massive public health significance. Iron deficiency anemia is one of the most frequent nutritional diseases reported in all over the world, but it is more common in the developing countries. Basic approach to control of IDA should comprise education and linked way to increase the dietary intake of iron, dietary modification to enhance the iron absorption, fortification of food articles, in addition to control the infection and worm infestations. Our present meta-analysis about the effect of dietary intervention on children with iron deficiency anemia established that dietary intervention had a beneficial effect on children with IDA, as supported by significantly increase effect of dietary intervention (OR = 5.03, 95% CI: 3.09- 8.18). In this paper, we do a meta-analysis with the literature retrieved over the past 36 years about dietary intervention role in IDA recovery and discuss the beneficial effect of dietary intervention on IDA recovery. Our result demonstrated that dietary intervention had a beneficial effect on children with IDA.

Tests for heterogeneity showed no significant differences across studies (χ2 = 1.97, *p* = 0.85), which could be explained the consistency in the China region. Meanwhile, studies involved in our meta-analysis were conducted in similar geographic regions, and the participants might share the different genetic background, lifestyle, dietary style, and so on. For the participants of all the included trials, those who take regular supplements, such as vitamin, or any nutrients that known to affect any variable determined were excluded according to their daily diet and dietary habits. Thus, our results provided certain evidences that dietary intervention might play a certain role in children with iron deficiency anemia.

Results from subgroup analysis indicated OR of 4 weeks intervention is higher. This may be ascribed to the short time, so that children with iron deficiency anemia can’t recover completely. Thus, our subgroup analysis provided some evidences that prolonged dietary intervention may be more effective in children with iron deficiency anemia. Our results from the sensitivity analysis showed that the pooled result was no affected by using a random effects model. Heterogeneity is the main factor our result from sensitivity analysis, thus more trials with large sample sizes are required in the future to eliminate the effect of heterogeneity and to confirm our results. In our results, dietary intervention can increase the clinical effect of children with iron deficiency anemia.

Several limitations in our current study should be addressed. First, only a small number of trials (*n* = 6), with a relatively small sample size, have been included in our study. Second, due to the small sample size, we failed to determine the role of clinical effect of dietary intervention in terms of intervention time. Third, tests for heterogeneity showed no significant differences, and the fixed effect model was employed. Because of the limited trials, we could not further analyze the effect of study design on the pooled results. Although the methodology is practical to combine the data from different study designs, more high quality trials are needed to confirm our findings.

There are several advantages to our study. Although only six studies were involved in our meta-analysis, it could provide relatively more statistical power and reliable estimates than individual studies to detect the association between dietary intervention and iron deficiency anemia in children. The original studies included in our final meta-analysis were all prospective, clinical intervention studies, which greatly reduced the likelihood of recall bias and selection bias. Especially, RCT provided much stronger support for a causal association than observation studies.

In our present study, our results, at least, testified that the dietary intervention had a beneficial effect on children with iron deficiency anemia. As a common disease among children, IDA is now a burden for both families and society. And in view of the side-effects of iron supplement, dietary intervention is now more popular and beneficial for children’s healthy. So our studies have important public health implications.

## Conclusions

In conclusion, our meta-analysis provides evidence of dietary intervention as effective additions for iron treatment. This may be due to the difference of study population, regional, diet usage, diet type and research methods. Therefore, a more rigorous scientific study is needed to continue to explore the beneficial effect of dietary intervention on IDA improvement.

## Abbreviations

CI: Confidence intervals
IDA: Iron deficiency anemia
OR: Odds ratio
